# Mechanisms inducing differentiation of adult islet progenitor-like cells into functional islet-like organoids

**DOI:** 10.3389/frtra.2026.1740314

**Published:** 2026-02-20

**Authors:** Carly M. Darden, Jayachandra Kuncha, Jeffrey T. Kirkland, Jordan Mattke, Srividya Vasu, Prathab Balaji Saravanan, Bashoo Naziruddin, Michael C. Lawrence

**Affiliations:** 1 Islet Cell Laboratory, Baylor Scott & White Research Institute, Dallas, TX, United States; 2 Annette C. and Harold C. Simmons Transplant Institute, Baylor University Medical Center, Dallas, TX, United States

**Keywords:** adult islet stem cells, beta-cell differentiation, chromatin remodeling, islet cell differentiation, islet cell replacement, islet progenitor cells, islet regeneration, islet-like organoids

## Abstract

**Introduction:**

Adult pancreatic tissue contains cell populations with latent regenerative potential, but the processes governing their expansion and differentiation into endocrine lineages remain unclear.

**Methods:**

Adult human pancreatic cells obtained from donor tissue were isolated and expanded and analyzed for lineage potential using single-cell RNA sequencing, flow cytometry, and functional assays. A CD9^+^ PROCR^+^ RGS16^+^ subpopulation, termed islet progenitor-like cells (IPCs), was evaluated for proliferative capacity and differentiation potential.

**Results:**

IPCs exhibited robust proliferative capacity and, upon differentiation, formed insulin- and glucagon-secreting organoids. Treatment of IPCs with the small molecule ISX9 induced expression of key transcription factors RFX6 and NEUROD1 through calcium-dependent chromatin remodeling mediated by NFAT recruitment of p300 and displacement of histone deacetylases (HDAC1-3). Pharmacologic inhibition of HDACs further enhanced IPC maturation and glucose-stimulated insulin secretion.

**Discussion:**

These findings define the molecular and epigenetic mechanisms driving the expansion and differentiation of adult IPCs into functional islet-like organoids, providing a foundation for future regenerative approaches using adult pancreatic tissue as a renewable source for endocrine cell replacement.

## Introduction

Islet transplantation offers a promising cell replacement therapy to prevent or reverse diabetes in individuals who have lost functional beta and alpha cells. However, the limited availability of donor islet tissue remains a major barrier to its broader clinical application. This limitation has driven intense investigation into alternative strategies, including *in vitro* islet cell engineering and beta-cell expansion to restore or maintain islet cell mass and function.

Significant progress has been made in generating stem cell-derived “beta-like” cells by directing their differentiation through stepwise protocols that mimic developmental processes. These protocols guide human embryonic stem cells (hESCs) or induced pluripotent stem cells (iPSCs) through definitive endoderm and pancreatic progenitor stages to ultimately produce glucose-responsive, insulin-secreting cells (1–3). Most strategies employ 4 to 7 sequential culture stages that activate key transcription factors critical to islet development, including *PDX1*, *NEUROG3*, *RFX6*, *NEUROD1*, *NKX2-2*, *NKX6-1*, *MAFA*, and *MAFB* (4–14).

Stem cell-derived islets (sc-islets) have demonstrated the ability to reverse diabetes in animal models and are currently being evaluated in phase I and II clinical trials (15). While their therapeutic potential is substantial, use of hESCs and iPSCs for treating type 1 diabetes (T1D) requires immunosuppression and raises concerns about tumorigenicity, oncogenic mutations, and ethical issues related to embryonic sources (16–18). As a result, alternative approaches using non-embryonic, non-genetically modified adult-derived cells, including chemically-induced iPSCs, are under active investigation (19–23).

Studies have shown that insulin- and glucagon-producing cells can be derived from pancreatic ductal epithelial cells and mesenchymal stromal/stem cells (MSCs) (24–36). However, these source populations are highly heterogeneous, and the specific cell subtypes capable of endocrine differentiation remain poorly defined. Even cultures purified for canonical MSC surface markers contain diverse subpopulations with varying differentiation potentials, influenced by both tissue origin and culture conditions. Moreover, it remains unclear whether these cells can directly give rise to insulin-producing cells or primarily support the function and proliferation of existing beta cells.

A longstanding question in the field is whether an adult human stem cell population exists that can generate functional, glucose-responsive beta cells. Some studies have provided evidence of *de novo* beta-cell regeneration in both animal and human models (37–39). Yet, other lineage tracing studies in mice have demonstrated that beta cells are no longer produced once existing insulin-producing cells are ablated (40). These findings have led to alternative hypotheses, including the possibility that beta-cell regeneration arises not from pluripotent precursors, but from dedifferentiated or partially differentiated beta cells retained in a progenitor-like state (41–46).

Supporting this idea, we have observed that stressed or cultured beta cells can undergo dedifferentiation, and under specific conditions, regain insulin production and secretory function *in vitro* (47). Based on these observations, we hypothesized that beta cells can revert to a progenitor state, enabling their expansion and redifferentiation into functional endocrine phenotypes. In this study, we identify and characterize a subpopulation of adult islet progenitor-like cells (IPCs) from adult human pancreas donor tissue and delineate the signaling and chromatin-regulatory mechanisms that induce their differentiation into functional islet-like organoids (hereafter referred to as islet organoids). These findings provide mechanistic insight into adult pancreatic plasticity and establish a foundation for future studies aimed at clinically scalable expansion and conversion of IPCs for autologous islet cell replacement therapies.

## Materials and methods

### Reagents and recombinant DNA constructs

Antibodies used were as follows: NFATC2, RGS16, NEUROD1, NEUROG3, p300, HDAC1, HDAC2, and HDAC3 (Santa Cruz Biotechnology, Inc); CD45-Alexa Fluor and CD29-Alexa Fluor (BioLegend); HLA Class 1 ABC (Abcam); and CD34-APC, CD105-APC, CD90-PE, CD73-PE (BD Biosciences). Gluc-ON reporters for RFX6 (HPRM53326-PG04), NEUROD1 (HPRM69533-PG04), and INS (HPRM30189-PG04) promoters were obtained from GeneCopoeia. Plasmid expression vectors dominant-negative NFAT PxIxIT motif (dnNFAT) and mutated dnNFAT AxAxAA motif (dnNFATm) were previously described (48–51).

### Cell and tissue culture

Adult human pancreatic tissue from multiple donors was obtained from research islet cell isolations performed in the cGMP Islet Cell Processing Laboratory at Baylor University Medical Center in accordance with institutional and national guidelines and regulations. Briefly, pancreata were enzymatically digested with collagenase and mechanically dissociated in a Ricordi chamber. Following enzyme dilution and washing, digested tissue was subjected to density gradient purification using a COBE 2,991 cell processor. Multiple fractions were collected during purification, each containing variable proportions of islet and non-islet pancreatic tissue. Fractions with lower islet purity and COBE bag remnant fractions, which are typically discarded after clinical isolation, were cultured *in vitro* under expansion conditions. Digested pancreatic tissue from multiple donors was obtained from the cGMP Islet Cell Processing Laboratory at Baylor University Medical Center in accordance with institutional and national guidelines and regulations. Pancreatic tissue and IPCs were washed, cultured, and expanded in RPMI 1,640 (Gibco) containing 11 mM glucose, 10% heat-inactivated fetal bovine serum, 10 mM HEPES (pH 7.4), 2 mM L-glutamine, 1 mM sodium pyruvate, 50 μM β-mercaptoethanol, 100 U/mL penicillin, and 100 μg/mL streptomycin at 37°C in 5% CO_2_ humidified air. IPCs were expanded to high clonal density for up to 28 days to produce IPC clusters. Analysis of IPCs and IPC clusters was performed on weekly passages 8–15. FBS was reduced to 2%–5% for ISX9-induced islet cell differentiation. IPCs and IPC clusters were cultured and differentiated in Nunc Lab-Tek II Chamber Slide Systems (Thermo Fisher Scientific) for immunofluorescent staining. Krebs-Ringer bicarbonate HEPES buffer media was used with low (2.8 mM) and high (16.7 mM) glucose for glucose-stimulated insulin secretion and high (50 mM) KCl for depolarization experiments. RPMI 1,640 with 2 mM L-glutamine, 1:200 ITS-X, 10 µM triiodo-L-thyronine (T3), 10 µM ALK5 inhibitor II, 10 µM ZnSO_4_, and 10 µg/mL heparin was used as a base medium for end stages 5–7 of sc-islet differentiation supplemented as follows: stage 5 endocrine progenitors (SANT-1, 0.05 µM retinoic acid, and 100 nM LDN193189 for 3 days), stage 6 immature sc-islets [100 nM gamma secretase inhibitor XX (GSiXX), and 100 nM LDN193189 for 7 days], and stage 7 mature sc-islets [n-acetyl cysteine (NAC), Trolox, and 2 μM R428 for 7d].

### Sc-RNA sequencing and analysis

IPCs were cultured in 10 cm culture dishes and washed twice with sterile, cold 1× phosphate-buffered saline (PBS). Cells were dissociated into single-cell suspensions using TrypLE Express (Gibco, no phenol red) at 37°C for 5–8 min and diluted 1:1 with PBS. Enzymatic activity was neutralized with complete culture medium (RPMI supplemented with 10% FBS), and cells were gently triturated to minimize clumping. The suspension was filtered through a 70 µm cell strainer and centrifuged at 2000 rpm for 5 min at 4°C. Pellets were resuspended in cold PBS containing 0.4% BSA. Cell counts and viability were determined using AO/PI staining on an automated cell counter. Suspensions were adjusted to 1,200 cells/µL with ≥90% viability and maintained on ice prior to loading onto the Chromium X platform for library preparation.

Human pancreatic IPCs were processed using the 10x Genomics Chromium Next GEM Single Cell 3′ Reagent Kit v3. Single-cell libraries were generated using Gel Bead-in-Emulsions and sequenced on an Illumina NextSeq 2,000 platform (P2-100 flow cell). Raw sequencing data were processed using the Cell Ranger Count pipeline (v7.1.0) aligned to the GRCh38-2020-A human reference genome.

Approximately 100,000 barcodes were retained following quality control filtering (200–82,150 UMIs; 200–8,749 detected genes; <20% mitochondrial transcripts). Automated reference-based annotation was performed using Azimuth (HuBMAP). Clustering and visualization were conducted using Loupe Browser with graph-based nearest-neighbor clustering, identifying 20 transcriptionally distinct clusters. Cluster-enriched marker genes were identified using differential expression analysis relative to all other clusters, and IPC-enriched clusters were defined based on coordinated expression of progenitor-associated transcripts.

### DNA transfection

Intact islet organoids were detached from tissue culture by 10 min incubation with TrypLE Express (Gibco) at 37°C and filtered prior to electroporation of ∼100 islet organoids per sample with 2 µg total DNA using a Neon Transfection System (ThermoFisher Scientific) for 2 pulses of 1,200 V for 20 ms. Islet organoids were cultured 24 h prior to experimental treatments. The Secrete-Pair Dual Luminescence Assay Kit (GeneCopoeia) was used to normalize for promoter-reporter transfection. Cells were then lysed in passive lysis buffer, and Firefly and Renilla luminescence was measured with a dual luciferase assay kit (Promega). Luminescence was measured using the Cytation5 Cell Imaging Multi-Mode Reader (BioTek).

### Diabetic nude mouse bioassay

NU/J (Nude mice, Jackson Laboratory, RRID:IMSR_JAX:002019, cat no. 002019, male, 8–12 wks old) between 24 and 28 g were rendered diabetic by intraperitoneal administration of 200 mg/kg body weight of STZ (Sigma). Mice were considered diabetic when fasting blood glucose was >200 mg/dL for 3 or more consecutive days. Mice were assigned to experimental groups using a randomization procedure to minimize selection bias. Each mouse was assigned a unique identification number and randomly allocated to either treatment or control group. Animals were anesthetized using inhaled isoflurane delivered via a calibrated vaporizer at concentrations ranging from 1%–3% during surgery and maintained at a flow rate of approximately 1 L/min until loss of pedal reflex and slowed respiration. Full-dose human islets (3,000 IEQ), marginal-dose human islets (1,500 IEQ), IPCs alone (1 × 10^6^), and marginal-dose islets with IPCs were transplanted into kidney capsules of diabetic nude mice. Slow release EthiqaXR analgesic was administered immediately post-surgery in order to provide strong pain relief with a safe respiratory profile that lasts several hours to reduce the need for handling mice and causing additional stress. Blood glucose and animal health were monitored daily. Mice were fasted for 6 h with water provided *ad libitum* prior to intraperitoneal administration of 20% glucose solution at 2 g glucose/kg body weight. Blood glucose was measured at 30-minute intervals for intraperitoneal glucose tolerance test (IPGTT) analyses. All animal surgeries and procedures were performed in accordance with approved institutional animal care and use committee protocols. In the diabetic experimental group, a subset of animals developed extreme hyperglycemia and associated clinical signs (e.g., weight loss, dehydration, lethargy) consistent with advanced diabetes. In accordance with our approved humane endpoints, these animals were euthanized to prevent unnecessary suffering. Euthanasia was performed by anesthetic overdose (5% isoflurane via vaporizer) followed by cervical dislocation to ensure death, in accordance with institutional and federal guidelines. The development of severe diabetes was an anticipated outcome of the experimental model, and all animals were closely monitored throughout the study. No unexpected adverse events occurred in the control group.

Nude mice were housed in a conventional facility maintained at 20–22°C with a 12-hour light-dark cycle. Mice were kept in individually ventilated cages (IVCs) with a maximum of five mice per cage. Cages contained autoclaved wood-chip bedding and animals had *ad libitum* access to sterilized food and water. Environmental enrichment, including nesting material and shelters, was provided to promote well-being.

### Whole-mount immunofluorescent staining

3D IPC-derived islet organoids and IPC monolayers were cultured on chamber slides. Culture medium was removed, and samples were gently washed with PBS. Cells were fixed with ice-cold 100% methanol for 5 min at −30 °C, followed by three washes in Wash Buffer (PBS containing 0.1% BSA and 0.1% Triton X-100). Cells were then blocked with blocking buffer (PBS containing 5% BSA and 0.1% Triton X-100) for 30 min at room temperature. Blocking buffer was removed and cells were incubated with primary antibody (1:100 dilution) prepared in dilution buffer (PBS containing 1% BSA and 0.1% Triton X-100) overnight at 4°C. Cells were then washed three times for 5 min in wash buffer and incubated with secondary antibody in dilution buffer for 1 h at room temperature. Following three additional washes, slides were mounted using VECTASHIELD® HardSet™ mounting medium. Samples were imaged and analyzed by an Olympus BX61 TRF Fluorescent Microscope.

### Flow cytometry

Cells were detached from the culture flask and dissociated into single cells by 10 min incubation in TrypLE Express (Gibco) at 37°C. Dissociated IPCs were filtered through a 40 µm sterile nylon cell strainer (Fisher Scientific), and single cells were then washed twice with FACS buffer (2% FBS in PBS). Viability and counting were performed using 0.4% Trypan Blue solution (Thermo Fisher Scientific). A total of 1 × 10^6^ cells in suspension were labeled with fluorochrome-conjugated surface antibody CD9. For intracellular staining of PROCR and RGS16, cells were fixed and permeabilized using Cytofix/Cytoperm (BD Biosciences) according to the manufacturer's instructions. FACS data were acquired on an LSR Fortessa flow cytometer with 5 lasers and 18 channels (BD Biosciences). Fluorescence minus one (FMO) cell sets and unstained cells served as biological negative controls. FACS data were analyzed by FlowJo software version 10.10.0.

### Chromatin immunoprecipitation (ChIP) assays

ChIP assays were performed as previously described (47). Islet organoids were fixed, and chromatin DNA-protein was cross-linked with 1% formaldehyde and sonicated with a Bioruptor 200 (Diagenode) to produce DNA fragments. DNA-protein complexes were immunoprecipitated with indicated antibodies or IgG isotype controls, extensively washed, and the cross-links were reversed by heating to 65°C for 4 h in Tris-HCl (pH 6.5), 5 M NaCl, and 0.5 M EDTA. DNA was extracted by phenol/CHCl3 and precipitated with ethanol. Precipitated DNA and 1% control inputs were analyzed by real-time quantitative polymerase chain reaction (qPCR).

### Real-time qPCR

Total RNA was isolated with the Qiagen RNeasy Mini Kit. Equal amounts of cDNA were synthesized using a High Capacity Reverse Transcription Kit (ThermoFisher). TaqMan primers were mixed with TaqMan Universal Master Mix II, with uracil N-glycosylase (ThermoFisher). 18S was amplified as an internal control. qPCR was performed using the Bio-Rad CFX connect system with TaqMan Primer Assays to detect 18S and target genes (ThermoFisher). RT^2^ qPCR primer assays were used to amplify NFATC2, PDX1, NEUROG3, RFX6, NEUROD1, NKX2-2, NKX6-1, MAFA, MAFB, SLC2A2, INS, and GCG genes (Qiagen). Primer sequences used to detect DNA input and immunoprecipitated 5′-flank promoter regions of human NEUROG3, RFX6, NEUROD1, and INS genes included RFX6 −499 to −274, 5ʹ- TGCAAAGACTGAGCGGTACT and 5ʹ- CCCTCCTTCCCGTTCTCTCA; NEUROD1 −516 to −70, 5ʹ-AGGCCACTCGCTCTGATCTA and 5ʹ-CTGAGGGGCTAGCAGGTCTA; NEUROG3, to −598 to −21, 5ʹ-TGGAAGGGACATAGGCAGGA and 5ʹ- CACGCTTTATCTGCTTCGCC; and INS −204 to −46, 5′-GTCCTGAGGAAGAGGTGCTG and 5′- CCATCTCCCCTACCTGTCAA.

### Insulin and glucagon secretion assays

Islet clusters pretreated with ITF2357 for 24 h and ISX9 for 14 days were isolated from cell cultures and incubated for 2 h in DMEM medium for recovery. Islet organoids were then transferred to low (2.8 mM) or high (16.7 mM) glucose in KRBH medium for 60 min before replacing and stimulating in static incubation conditions with high (for insulin release) or low (for glucagon release) glucose KRBH medium for 60 min, respectively. Insulin and glucagon protein from cells and supernatant were measured by Insulin (ALPCO) and Glucagon Quantikine (R&D Systems) ELISA kits, respectively.

### Statistical methods

Statistical analysis was performed using GraphPad Prism version 9.0 (La Jolla, CA). Statistical significance for more than two groups was determined by two-way analysis of variance and Sidak's multiple comparison test. Direct comparisons between two experimental groups were determined using unpaired Student's *t* test. Differences were considered significant when *p* values were <0.05 (*), <0.01 (**), and <0.001 (***).

## Results

### Single cell RNA-seq analysis of expanded pancreatic tissue reveals a subpopulation of islet progenitor cells in a dedifferentiated state

We used transcriptomic profiling to analyze single cells from cultured human pancreatic tissue to identify and characterize progenitor cells expanded in culture. Adult pancreatic samples were collected from islet cell purification fractions during islet isolation procedures and expanded in culture for 8–15 passages (Supplementary Table S1). Single-cell RNA sequencing was then performed on dissociated cells to profile the expanded populations. A total of 100,000 cell barcodes were processed and analyzed using reference-based mapping against integrated human pancreatic tissue scRNA-seq datasets from six independent studies using various single-cell technologies compiled in the Human BioMolecular Atlas Program (HuBMAP) (52, 53)*.*

**Figure 1:**
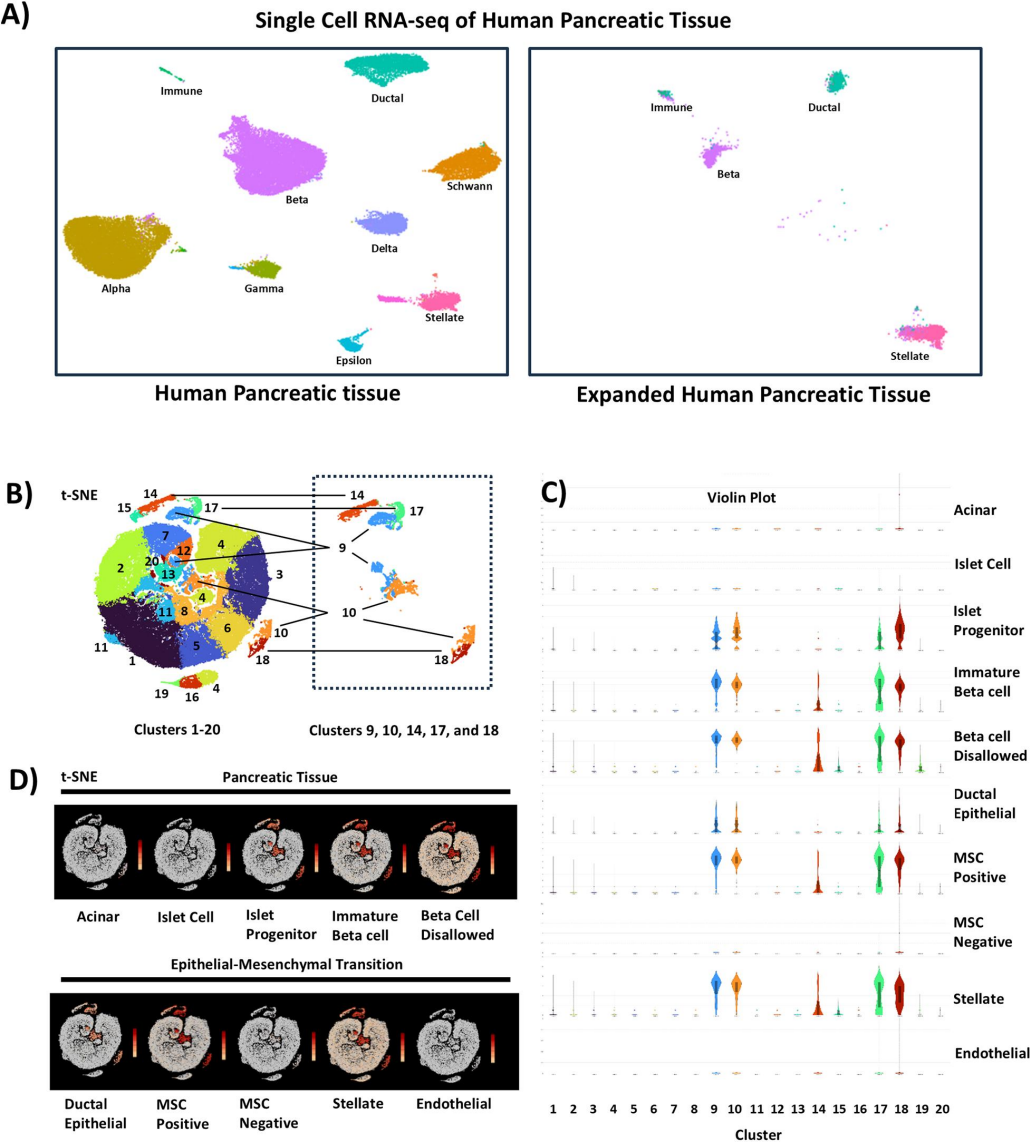
Identification of IPCs in expanded human pancreatic tissue by single cell transcriptomic profiling. **(A)** Azimuth automated cell type annotation mapping of scRNA-seq data from expanded human pancreatic tissue (100,000 cells) referenced to dataset aggregates (*n* = 6) from human pancreatic tissue (35,000 cells) and **(B)** Seurat analysis of highly variant genes and dimensionality reduction by t-distributed stochastic neighbor embedding (t-SNE). **(C)** Gene expression profiling and **(D)** t-SNE mapping of cluster 9, 10, 14, 17, and 18 subpopulations containing islet cell progenitor cell markers (9,289 cells). Data shown are results from one donor.

Automated cell type annotation using Azimuth identified multiple pancreatic cell subtypes with transcriptomic signatures matching activated stellate cells, ductal cells, and beta cells (Figure 1A) (54). Clustering and visualization were performed using Loupe Browser (10x Genomics), which applies graph-based clustering following dimensionality reduction. This analysis identified 20 transcriptionally distinct clusters (Figure 1B). Clusters 9, 10, 14, 17, and 18 were selected based on differential gene expression relative to all other clusters and coordinated expression of endocrine progenitor–associated transcripts, together with reduced expression of mature endocrine, acinar, and endothelial markers (Figures 1B–D; Supplementary Table S2). Marker enrichment within clusters was determined using differential expression analysis implemented in Loupe Browser.

Importantly, this subpopulation lacked expression of mature endocrine, acinar, or endothelial markers while expressing transcripts associated with immature beta-like states and beta cell-disallowed genes. These transcriptional features are consistent with a progenitor-like or partially dedifferentiated endocrine phenotype.

### IPCs express multiple progenitor markers and signaling pathways associated with regeneration

**Figure 2:**
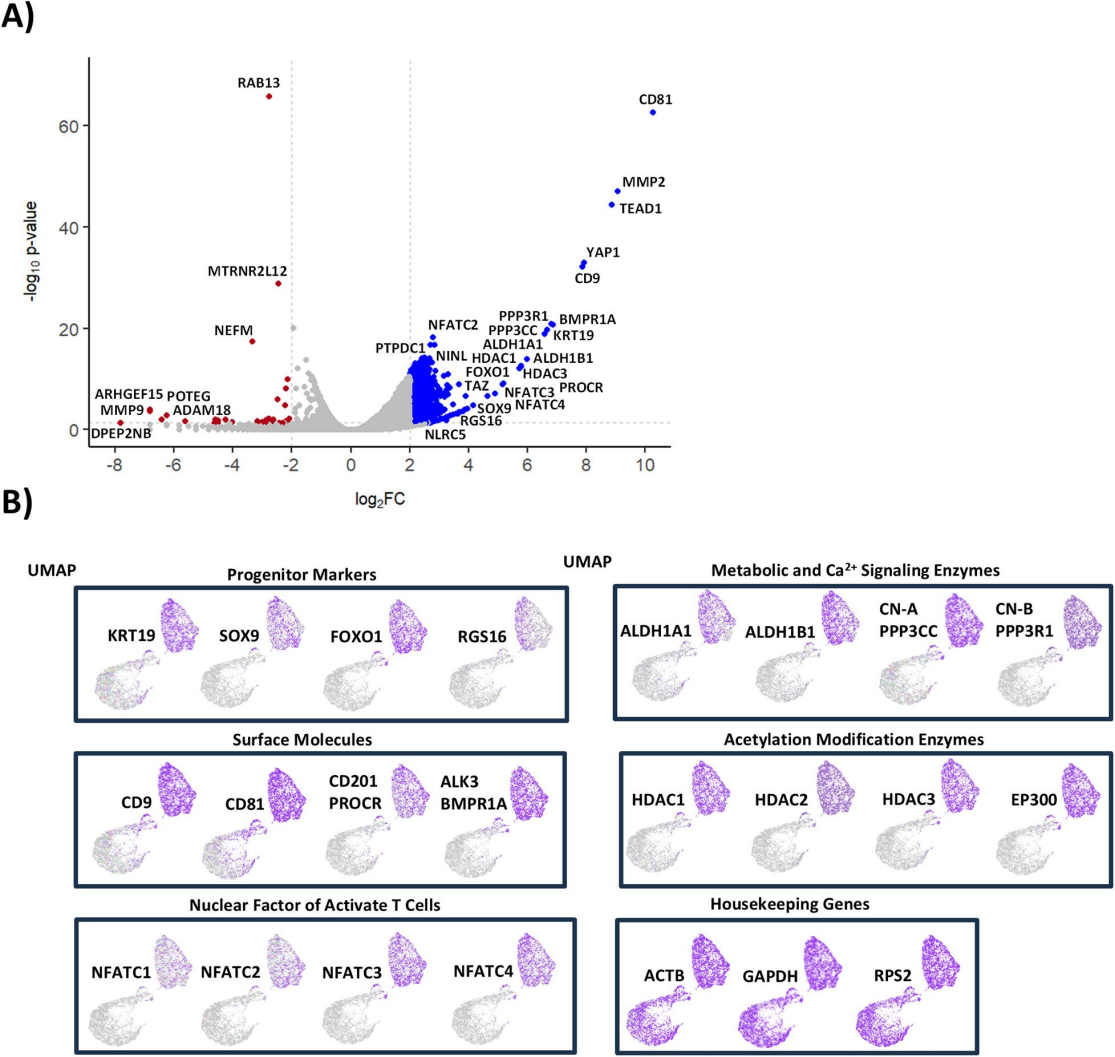
Gene enrichment analysis of IPCs in expanded human pancreatic tissue. **(A)** Differential gene expression analysis and **(B)** Uniform Manifold Approximation and Projection (UMAP) analysis of IPCs. Data shown are results from one donor.

Further reclustering and differential gene expression analysis of the IPC population revealed strong upregulation (>50-fold) of transcripts including *CD81*, *MMP2*, *TEAD1*, *YAP1*, *CD9*, *KRT19*, *BMPR1A*, *PPP3R1*, *PPP3CC*, *ALDH1A*, *ALDH1B1*, *HDAC1*, *HDAC3*, and *FOXO1* (Figure 2A). Gene set enrichment analysis (GSEA) of the top 100 upregulated genes showed highest enrichment in pancreatic progenitor cell types, matched against the PanglaoDB and CellMarker 2.0 databases (Supplementary Table S3) (55, 56). The most enriched signaling pathways included those associated with epithelial-mesenchymal transition (EMT) and beta-cell development, based on matches to the MSigDB Human Molecular Signatures database (Supplementary Table S4A) (57). Gene Ontology analysis of Molecular Function revealed strong enrichment in *ALDH* activity and *BMP receptor* signaling (Supplementary Table S4B) (58, 59).

Uniform Manifold Approximation and Projection (UMAP) highlighted selective expression of progenitor genes *KRT19*, *SOX9*, *FOXO1*, and *RGS16*, enriched in one of two major IPC subpopulations (Figure 2B). This cluster also co-expressed surface molecules (*CD9*, *CD81*, *PROCR/CD201*, *BMPR1A*), metabolic regulators (*ALDH1A*, *PPP3CC*), histone modification enzymes (*HDAC1-3*, *EP300*), and NFAT family transcription factors (*NFATC1–C4*).

The gene enrichment analyses suggest that the expanded IPC population is in a state of EMT, as previously shown to be a characteristic of an islet cell progenitor state (60–62). Moreover, IPCs selectively express surface markers previously identified in pancreatic endocrine progenitor cells and immature beta cells, including CD9, CD81, PROCR, and BMPR1A (34, 63–67). The IPCs are enriched with ALDH1 isozymes 1A1 and 1B1, which were shown to be associated with regulation of differentiation of endocrine progenitor cells (68, 69). High relative expression of histone modification enzymes p300 and HDAC family members indicates the cells' potential capability to regulate genes at the chromatin level. The IPCs are also enriched with calcium/calmodulin dependent phosphatase calcineurin (CN) A and B subunits and downstream transcription factor targets of the NFAT family, suggesting potential capacity to modulate genes in response to calcium signaling. This is in line with previous observations that beta cells utilize CN/NFAT signaling to maintain beta-cell differentiation, mass, and function (47, 50, 70–75). Altogether, these data indicate that the expanded IPCs express gene profiles with islet endocrine progenitor characteristics and the potential capacity to support islet cell regeneration and function.

### Expanded IPCs enhance islet graft function *in vivo*

Because expanded IPCs exhibited gene expression profiles suggestive of islet regenerative capacity, we examined their potential to provide supportive effects *in vivo* when combined with islet grafts. Expanded human IPCs were co-transplanted with subtherapeutic (marginal) doses of human islets into streptozotocin-induced diabetic nude mice. Co-transplantation resulted in improved glycemic control compared with marginal islets alone, as assessed by longitudinal blood glucose monitoring and intraperitoneal glucose tolerance testing (Supplementary Figures S1A–C). Following nephrectomy of the graft-bearing kidney, a subset of co-transplanted animals exhibited transient residual glycemic effects.

### CD9^+^, PROCR^+^ IPCs form RGS16^+^ IPC clusters with the capacity to differentiate into islet organoids *in vitro*

**Figure 3:**
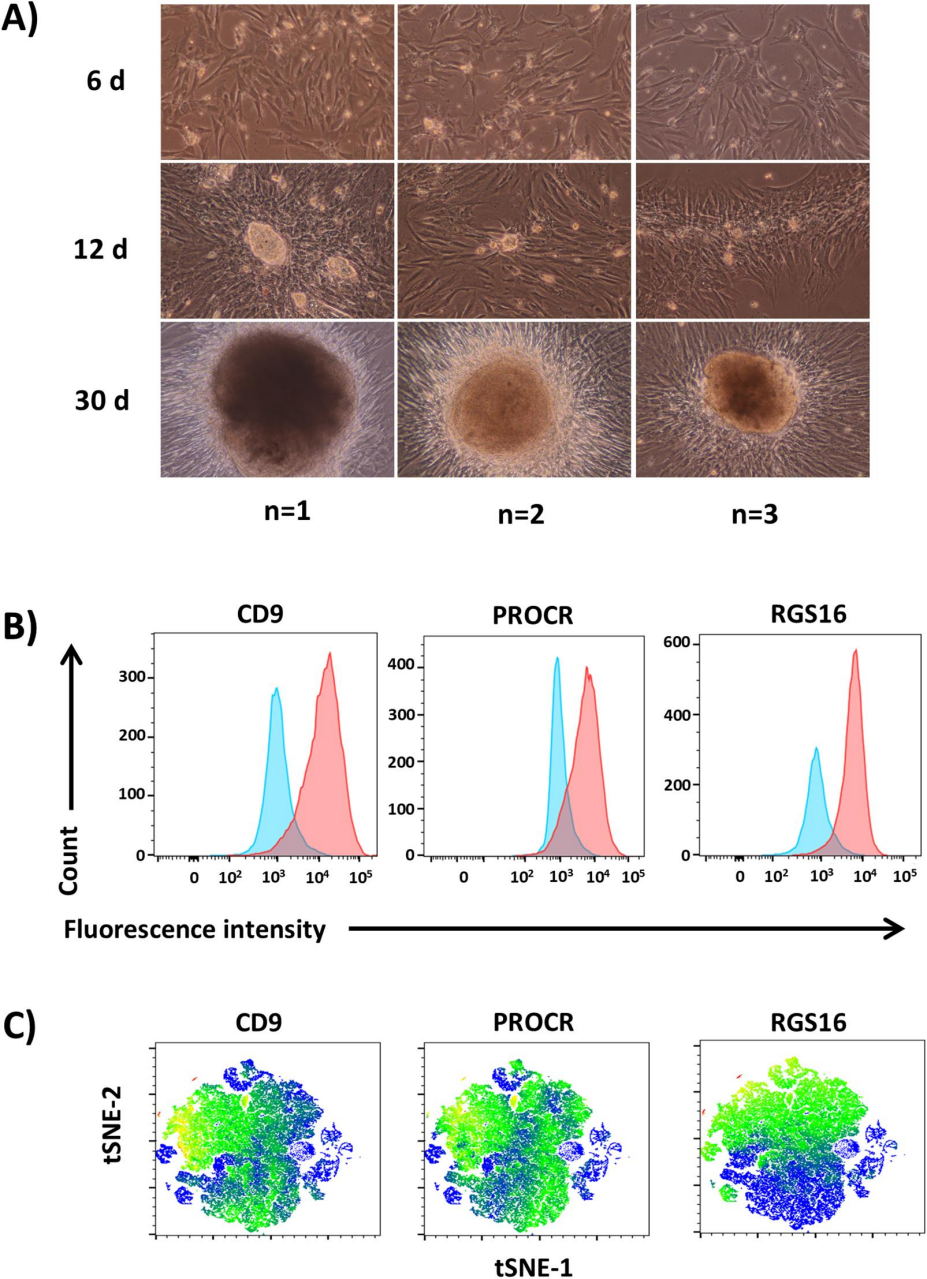
Characterization of IPCs and formation of IPC clusters. **(A)** Micrograph brightfield (200×) images of expansion of IPCs and formation of IPC clusters within 30 days of culture. Flow cytometry analyses of pancreatic progenitor and beta cell dedifferentiation surface markers CD9 and PROCR and internal islet endocrine lineage marker RGS16 expressed in IPCs by **(B)** counts and fluorescent intensity and **(C)** t-distributed stochastic neighbor embedding (t-SNE) heat mapping of CD9, PROCR, and RGS16 protein expression. Data shown are representative results from at least three independent experiments.

**Figure 4:**
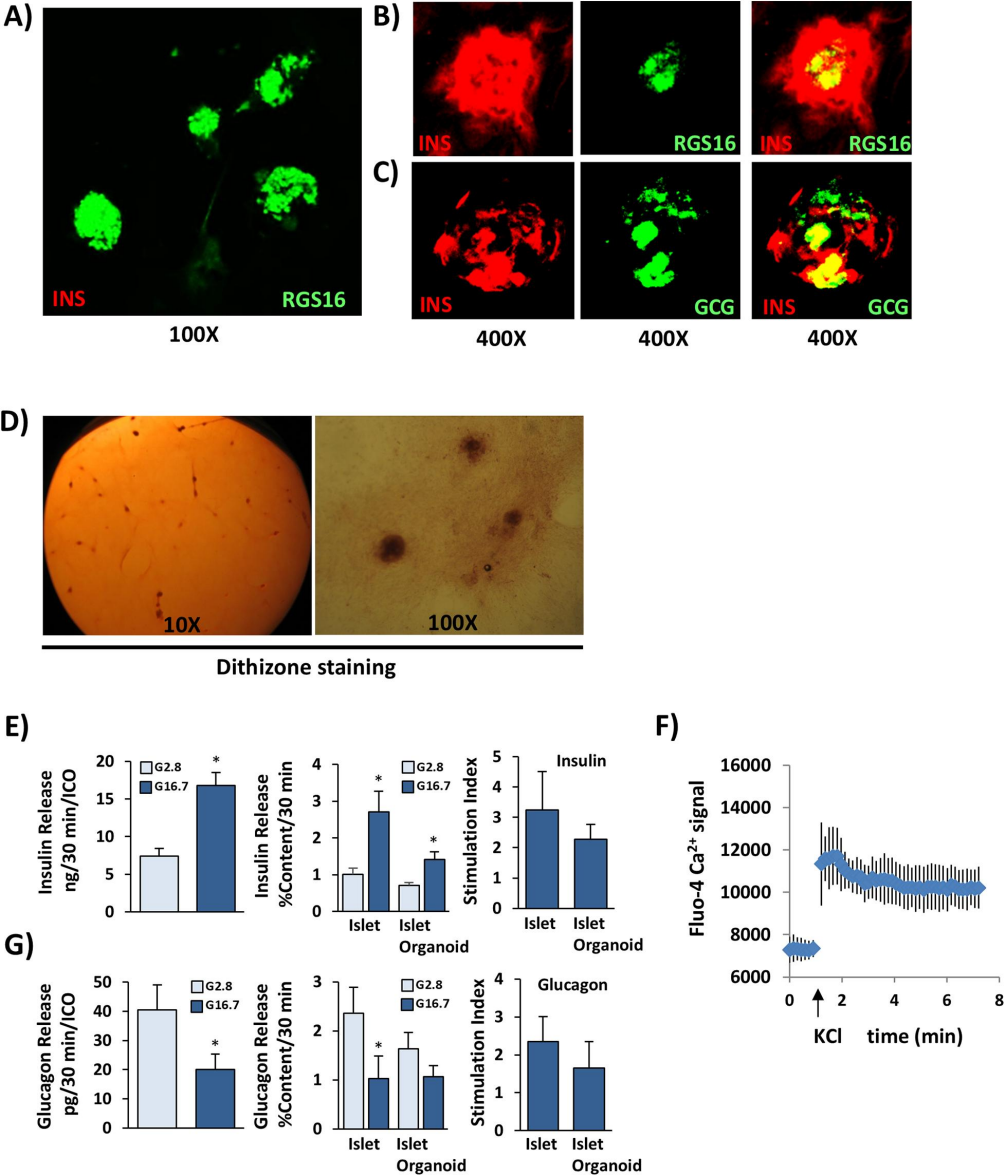
Differentiation of RGS16^+^ IPC clusters into islet organoids by ISX9. Immunofluorescent staining of RGS16 and islet hormones INS and GCG in human IPCs treated with **(A)** DMSO (control) and **(B,C)** ISX9 for 14 days *in vitro* to assess for **(B)** INS (red)/RGS16 (green) and **(C)** INS (red)/GCG (green) cellular co-localization. **(D)** Dithizone staining of IPC organoids treated with ISX9 for 14 days. **(E)** Glucose-stimulated insulin secretion assay comparing human islet organoids to normal isolated islets. **(F)** Assessment of intracellular calcium release in islet organoids by KCl-induced depolarization. **(G)** Glucagon release in islet organoids in response to high (25 mM) and low (2.5 mM) glucose. Graphed values are expressed as mean ± SD. Asterisks indicate statistically significant differences low- and high-glucose conditions within each group (*p < 0.05, **p < 0.01, ***p < 0.001; two-tailed Student's *t*-test). Absence of an asterisk indicates no statistically significant difference. Data shown are representative of at least three independent experiments.

Previous studies have indicated that various sources of adult human tissues can differentiate into insulin-producing cells *in vitro* (25, 28, 31, 43, 44, 76–78). Thus, we sought to determine if expanded IPCs have the potential to differentiate and become glucose-responsive islet-like cells *in vitro*. Dissociated IPCs formed monolayers that expanded into cellular networks and formed dense islet-like cell clusters upon reaching high density within 30 days of culture (Figure 3A). To define markers for further characterization of IPCs with potential islet regenerative properties, we performed flow cytometry to confirm expression of surface molecules identified in our transcriptomic analysis of highly differentially expressed genes clustering with islet progenitor, immature beta cell, and beta-cell disallowed genes (Figures 1, 2A). Flow cytometry analysis confirmed co-expression of CD9 and PROCR surface proteins on expanded IPCs (Figure 3B). The IPCs also internally expressed islet cell progenitor protein RGS16 corresponding to the single cell RNA-seq data, indicating its clustering with islet cell progenitor genes. RGS16 was previously identified as a marker for early pancreatic progenitors that is temporally expressed in IPCs prior to terminal maturation (79, 80). Dimensional reduction analysis heat mapping of protein expression by t-SNE confirmed high co-expression of CD9 and PROCR surface molecules overlapping with a large subset of IPCs expressing RGS16 (Figure 3C). Immunofluorescent staining showed RGS16 was selectively expressed in the IPC clusters of expanded IPCs which were devoid of insulin (Figure 4A). Overall, the results suggested that CD9^+^PROCR^+^ IPCs expand and form IPC clusters containing RGS16^+^ islet-like cells in a dedifferentiated or progenitor state.

Our transcriptomic analysis indicated that CD9, PROCR, and RGS16 genes also clustered with cells expressing NFAT and HDAC signaling enzymes. We previously showed that small molecule differentiator isoxazole-9 (ISX9) could restore beta-cell function and prevent dedifferentiation by NFAT-mediated regulation of p300 and HDAC of beta-cell differentiation and disallowed genes at the chromatin level (47). Thus, we hypothesized that ISX9 could likewise induce differentiation or maturation of expanded IPCs that retained this signaling system. Treatment of expanded IPCs with ISX9 for 14 days resulted in selective expression of insulin and glucagon within islet organoids as determined by immunofluorescent staining (Figures 4B,C). The surrounding IPC monolayer remained largely unstained (Supplementary Figure S3). Notably, residual RGS16 expression was observed in the inner core of large islet organoids, suggesting that some of the core cells were not completely differentiated (Figure 4B). Likewise, some core organoid cells co-expressed both insulin and glucagon, indicating that these islet-like cells remained in an immature state (Figure 4C). However, most insulin-expressing organoid cells no longer expressed RGS16, and a large portion of the islet organoid cells expressed insulin and glucagon independently. Moreover, the islet organoids also intensely stained burgundy red with diphenylthiocarbazone (dithizone), suggesting enrichment of zinc-containing insulin granules, which are produced by mature beta cells (Figure 4D).

To assess functional maturity of islet organoids, we tested their ability to release insulin and glucagon in response to glucose. Indeed, the islet organoids released insulin in response to high glucose with stimulation indices comparable to those of freshly isolated human islets (Figure 4E). They also exhibited K^+^-induced depolarization as determined by calcium influx in response to 30 mM K^+^ (Figure 4F), suggesting functional K_ATP_ channels as expressed in mature beta cells. Furthermore, the islet organoids showed increased glucagon release in response to low glucose, indicative of alpha-like cell function (Figure 4G). These data indicate that the RGS16^+^ IPC clusters can be differentiated into functional glucose-responsive islet organoids within 14-day treatment with ISX9 *in vitro*.

### ISX9 induces sequential activation of islet-lineage transcription factors that drive alpha- and beta-cell differentiation

**Figure 5:**
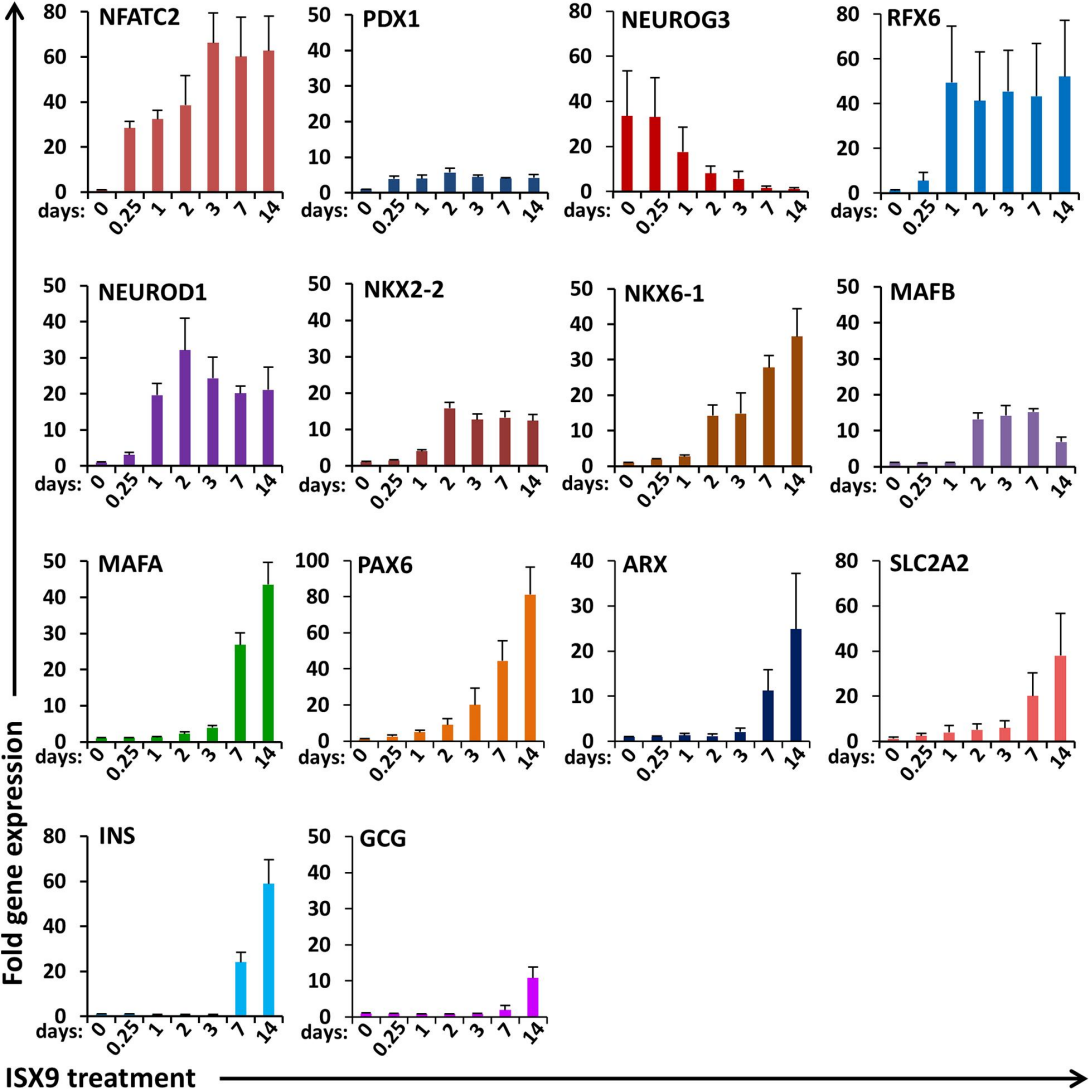
Time-course analysis of islet cell lineage genes expressed during IPC islet organoid differentiation. Expression of mRNA for transcription factors involved in islet cell differentiation and maturation in IPCs treated with ISX9 for up to 14 days. Graphed values are expressed as mean ± SD. Data shown are results from at least three independent experiments.

To identify transcription factors that may contribute to islet organoid differentiation from IPCs in culture, we tracked expression of genes known to be determinants of islet endocrine lineages during defined stages of islet cell development. Time-course analysis showed NFATC2 to be one of the earliest genes expressed within 6 h of ISX9 stimulation (Figure 5). Early endocrine progenitor gene NGN3 was reduced and often undetectable within 14 days of ISX9 treatment. In contrast, islet differentiation transcription factors RFX6 and ND1 were highly inducible within 24 h, followed by induction of NKX2.2 and NKX6.1 occurring within 48 h. Beta-cell and alpha-cell restricted transcription factors MAFA and MAFB were expressed within 2 and 7 days, respectively. PAX6 and ARX, which are associated with differentiation of alpha cells, were progressively expressed alongside induction of beta-cell-lineage transcription factors. Finally, insulin and glucagon along with the SLC2A2 (GLUT2) transporter gene expression were detected within 7–14 days upon ISX9 treatment. These data indicate that IPC islet organoid differentiation *in vitro* recapitulates gene programming similar to what is observed during islet endocrine cell specification in stem cells and developing islets.

### CN and NFAT signaling is required to induce RFX6 and NEUROD1 gene expression in differentiating IPC islet organoids

**Figure 6:**
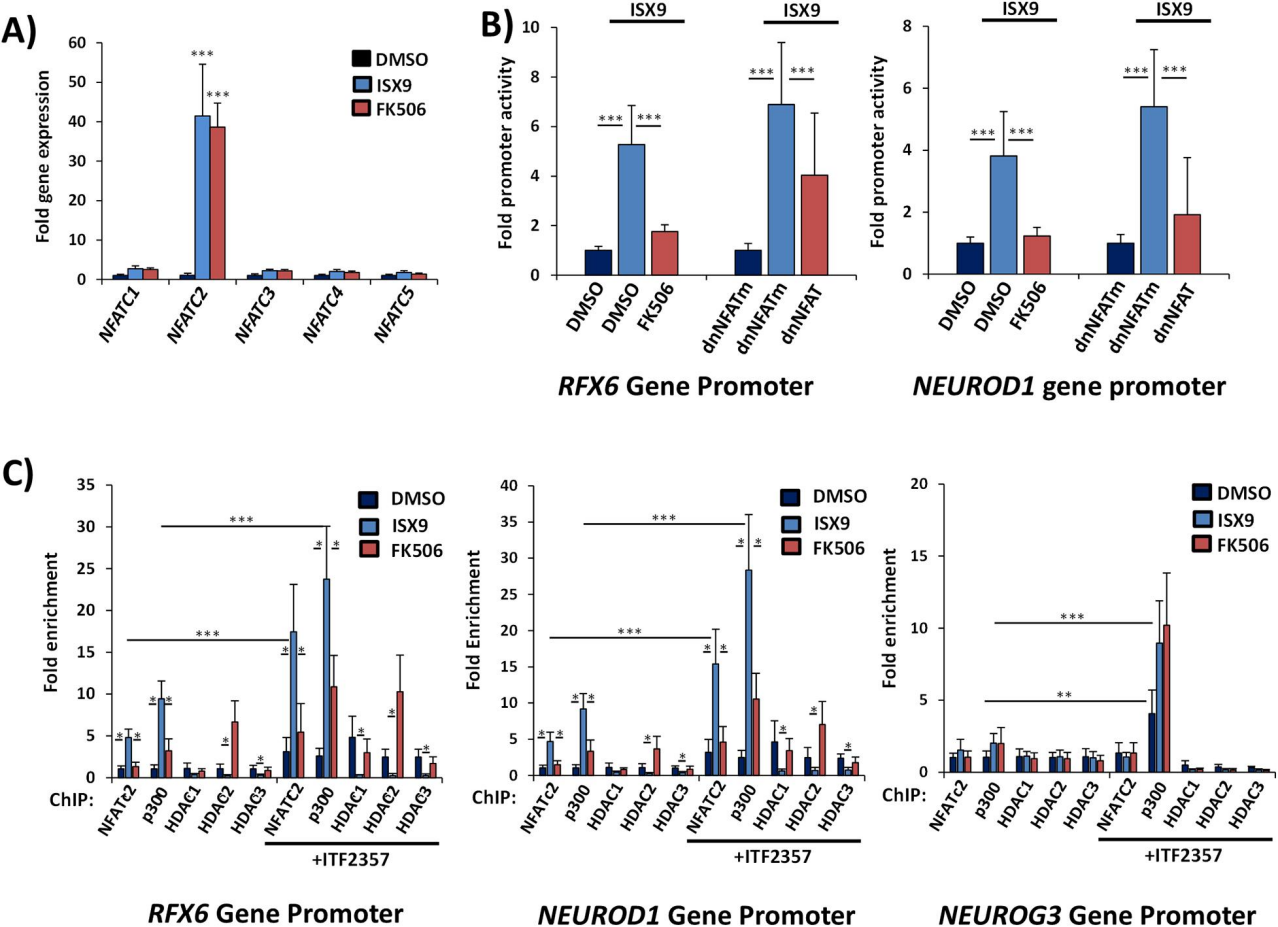
Requirement of CN/NFAT signaling to recruit p300 to RFX6 and NEUROD1 promoters during ISX9-induced IPC islet organoid differentiation **(A)** induction of NFAT family isoform genes in IPC clusters treated with ISX9 for 24 h in the presence of CN inhibitor FK506. **(B)** RFX6 and NEUROD1 promoter activation by ISX9 in IPCs in the presence of FK506 or transfected with gene vectors overexpressing dominant negative NFAT (dnNFAT) and mutated control (dnNFATm). **(C)** ChIP assay of association of NFATC2, p300, HDAC1, HDAC2, and HDAC3 with RFX6, NEUROD1, and NEUROG3 promoters upon 6 h treatment of IPCs with ISX9 with and without 24 h pretreatment with ITF2357. Graphed values are expressed as mean ± SD. Asterisks above bars indicate statistically significant differences (*p < 0.05, ***p < 0.001) in mean values for treatments based on a two-way ANOVA and Sidak's multiple comparison test. Data shown are results from at least three independent experiments using IPCs derived from three individual donors.

RFX6 and NEUROD1 have been shown to be the earliest transcription factors that promote differentiation of endocrine progenitors into islet-specific cell lineages. Both transcription factors are also required to maintain beta-cell identity, maturation, and functional state (81–84). Because ISX9 was previously shown to modulate calcium and CN/NFAT signaling in islet cells, we sought to determine if NFAT was required for activation of RFX6 or NEUROD1 genes during organoid differentiation. ISX9-induced differentiation of IPC organoids correlated with the rapid induction of NFATC2 prior to expression of RFX6 and NEUROD1 in comparison to other NFAT isoforms (Figures 5, 6A). Selective induction of NFATC2 expression by ISX9 was independent of CN activity, as it was insensitive to CN inhibitor FK506. Promoter-reporter assays showed increased RFX6 and NEUROD1 gene promoter activity after 24 h treatment with ISX9 (Figure 6B). Induction of RFX6 and NEUROD1 promoters was blocked by CN inhibitor FK506 or overexpression of a dominant negative NFAT (dnNFAT) protein containing a truncated PxIxIT box motif as compared to DMSO and mutated dnNFAT controls. These data indicate that ISX9 induced expression of both RFX6 and NEUROD1 at the gene promoter level in IPC organoids in a CN- and NFAT-dependent manner.

### HDAC inhibition and activation of CN/NFAT signaling induces insulin gene expression in adult IPCs

We previously showed that calcium-dependent CN/NFAT is required for beta-cell differentiation and insulin gene transcription (47). Small molecule differentiation inducer ISX9 was able to prevent dedifferentiation of stressed islet beta cells by preserving intracellular calcium signaling and CN/NFATc2-mediated induction of beta-cell transcription factors and repression of disallowed genes. In contrast, extended exposure of islets to stress resulted in loss of CN/NFATc2 signaling, accumulation of HDACs on the RFX6 gene promoter, and beta-cell dedifferentiation. Thus, we hypothesized that inhibiting HDACs and reactivating CN/NFAT in IPC clusters would promote their differentiation into islet organoids.

To test this hypothesis, we pretreated IPCs with HDAC inhibitor ITF2357 for 24 h prior to treatment with ISX9 or inhibition of NFAT deactivating kinase Dyrk1a by harmine. Insulin gene transcription was induced within 10 days of exposure to both ISX9 and harmine, which in each case was accentuated by pretreatment with ITF2357 (Supplementary Figure S2). Both ISX9 and harmine showed enhanced effects to induce insulin expression in IPCs compared to treatment with modified end-stage differentiation protocols commonly used to stimulate endocrine cell progenitor stage to mature sc-islets (stages 5–7) in hESCs and iPSCs. These results suggest that CN/NFAT signaling can be targeted to pro-differentiate adult IPCs with relatively high proficiency.

### NFATc2 mediates chromatin assembly of histone acetylation-modifying enzymes p300 and HDACs on the RFX6 and NEUROD1 gene promoters

To determine if NFATC2 directly binds to the RFX6 gene promoter, we performed chromatin immunoprecipitation (ChIP) assays on islet organoids cultured in ISX9 for 24 h. ChIP analysis revealed significant increases in enrichment of NFATC2 on the 5ʹ flanking region of the RFX6 and NEUROD1 gene promoters (Figure 6C). In contrast, ISX9 had no effect on NFATC2 binding to the upstream endocrine progenitor transcription factor NEUROG3. Association of NFAT with the RFX6 and NEUROD1 promoters was largely prevented by FK506, indicating the requirement of CN for NFAT-mediated transcription. Previous studies showed that ISX9 could enhance p300 acetyltransferase recruitment to the insulin gene promoter to acetylate histones in MIN6 beta cells (41). We therefore sought to determine effects of ISX9 to target chromatin-related proteins to the RFX6 and NEUROD1 gene promoters in islet organoids. ChIP analysis was performed on ISX9-treated islet organoids to assess DNA promoter assembly of NFATC2 and histone-modifying enzymes p300 and HDACs. NFATC2 association correlated with accumulation of p300 upon the RFX6 and NEUROD1 promoters within 6 h of ISX9 treatment (Figure 6C). By contrast, HDACs were depleted on the RFX6 and NEUROD1 promoters under conditions that stimulated NFATC2 binding. In each case, FK506 inhibited effects of ISX9 to promote p300 and diminish HDAC accumulation on the RFX6 promoter. In contrast, ISX9 and FK506 did not affect association of p300 or HDACs with the NGN3 promoter, indicating that ISX9-induced CN/NFAT signaling mediates specificity of p300 recruitment toward the RFX6 and NEUROD1 genes (Figure 4D). Pretreatment of IPC clusters with HDAC inhibitor ITF2357 (50 nM) enhanced the effects of ISX9 to recruit p300 to the RFX6 and NEUROD1 promoters and also induced a significant increase in the ratio of p300:HDAC accumulation on the NEUROG3 promoter. FK506 blocked effects of ITF2357 on the RFX6 and NEUROD1 promoters, but p300 and HDAC accumulation on the NGN3 promoter were unchanged. Increases in p300:HDAC ratios correlated with increased gene expression. Whereas ISX9 selectively induced transcriptional activity of the RFX6 and NEUROD1 genes, ITF2357 globally promoted transcription from all genes. The results delineate CN/NFAT-mediated displacement of HDACs by p300 to selectively activate the RFX6 and NEUROD1 genes in response to ISX9 and a global effect of ITF2357 to increase the ratio of p300:HDAC promoter accumulation on genes to enhance induction of transcriptional activity. These data suggest that CN/NFATc2-mediated recruitment of p300 and induction of the RFX6 and NEUROD1 genes bypasses requirements of NGN3 to promote differentiation of IPCs to an islet-like phenotype.

### IPC-derived islet organoids are glucose- and GLP-1-responsive

**Figure 7:**
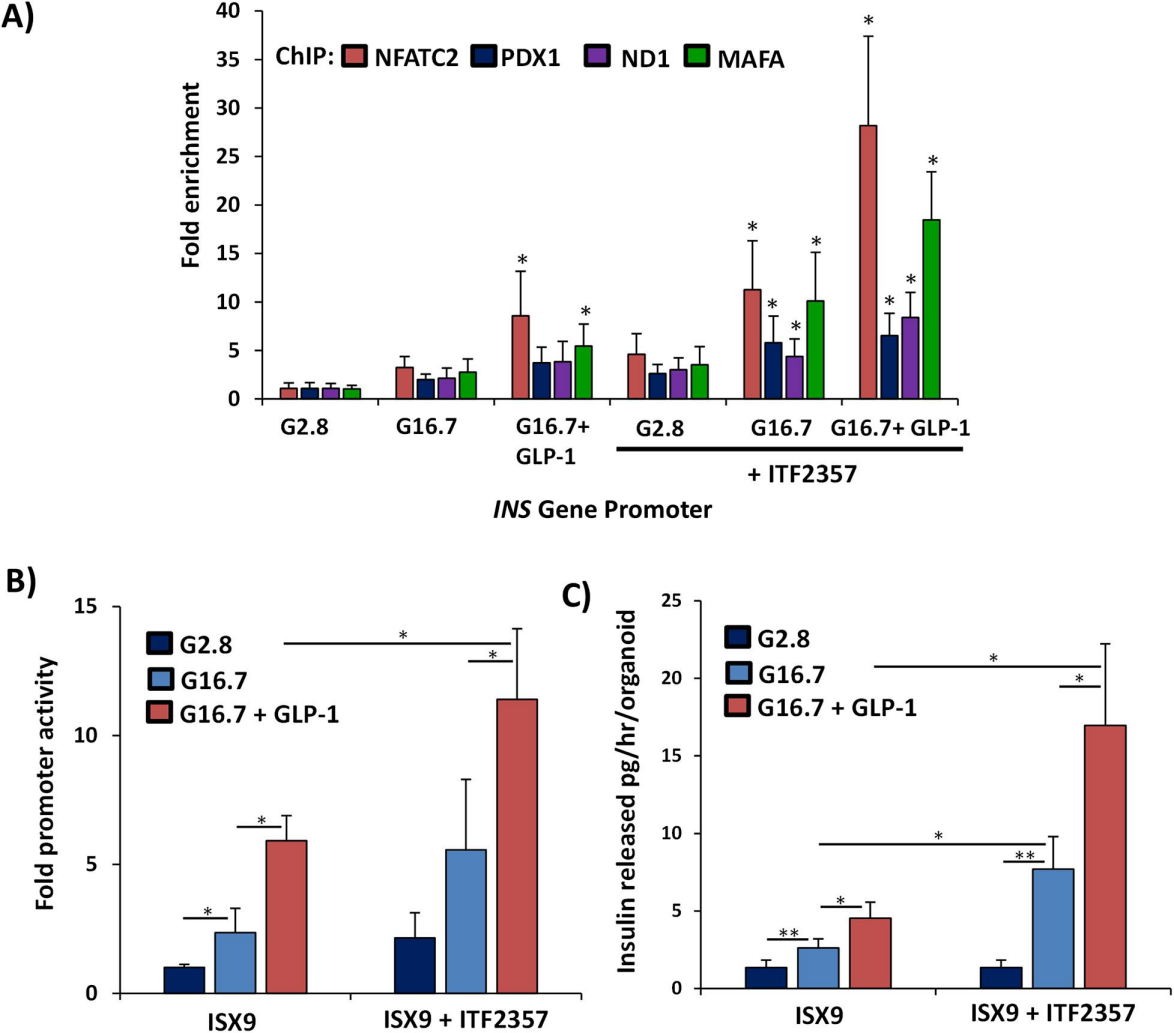
Glucose and GLP-1 responsiveness of differentiated IPC islet organoids. **(A)** ChIP assay of association of NFATC2, PDX1, ND1, and MAFA in islet organoids in response to 20 min treatment with high glucose (G16.7) and GLP-1. **(B)** Insulin-promoter activity in response to 24 h treatment with high glucose (G16.7) and GLP-1. **(C)** Glucose-stimulated insulin secretion assay of islet organoid insulin secretion in response to 2 h treatment with high glucose (G16.7) and GLP-1. Graphed values are expressed as mean ± SD. Asterisks above graphs indicate statistically significant differences (*p < 0.05) in mean values for treatments compared to 2.8 mM glucose controls based on a two-tailed Student's *t* test. Asterisks above bars indicate statistically significant differences (*p < 0.05, **p < 0.01) in mean values for treatments based on a two-way ANOVA and Sidak's multiple comparison test. Data shown are results from at least three independent experiments using islet cultures derived from three individual donors.

To release appropriate and sustainable insulin in response to physiological demand, beta cells must regulate insulin production in response to changes in glucose and nutrient-responsive incretins including peptide hormone glucagon-like peptide 1 (GLP-1). Therefore, we assessed effects of high (16.7 mM) glucose and GLP-1 on insulin gene expression and output in IPC-derived islet organoids (Figure 7). ChIP analysis of the insulin promoter demonstrated binding of key transcription factors PDX1, NEUROD1, and MAFA known to regulate insulin gene transcription in mature beta cells (Figure 7A). NFATC2 and MAFA showed highest enrichment upon the insulin gene promoter in response to glucose and GLP-1. The effect of NFATC2 and MAFA was enhanced along with significant enrichment of PDX1 and NEUROD1 upon the insulin gene promoter when IPC clusters were primed for 24 h with ITF2357 prior to 24-hour treatment with ISX9. Islet organoids transfected with a luciferase insulin gene promoter-reporter showed upregulation of the insulin gene in response to glucose and GLP-1, corresponding to increased binding of NFATc2 with PDX-1, NeuroD1, and MafA to the insulin gene promoter (Figures 7A,B). Insulin promoter activity was stimulated more than 5-fold in the presence of high glucose and GLP-1, which was enhanced to more than 10-fold when IPC clusters were primed with ITF2357. To test effects of glucose and GLP-1 on insulin secretion, we performed glucose-stimulated insulin secretion assays on IPC-derived islet organoids. High glucose treatment of islet organoids for 2 h resulted in insulin release of 7.7 ± 2.1 pg/h/organoid and 17.0 ± 5.3 pg/h/organoid in the presence of GLP-1 (Figure 7C). Overall, these data indicate that differentiated islet organoids produce and release insulin in response to glucose and GLP-1 by mechanisms similar to those observed in mature pancreatic beta cells.

## Discussion

A large body of evidence over the past century has indicated that the pancreas possesses regenerative capacity for both exocrine and endocrine tissue (85–91). Several models of pancreatic injury have shown that repair and restoration are facilitated in part by stem cell progenitors, which are capable of regenerating pancreatic tissue (85–92). However, the identity of adult islet progenitor cells remains unresolved, primarily due to the lack of specific markers for defining or tracking such populations. Moreover, the notion that adult stem/progenitor cells contribute to islet regeneration has been controversial, as lineage tracing studies in transgenic mice have suggested that increases in beta-cell mass after birth occur primarily through replication of pre-existing beta cells (40).

In this study, we identified and characterized expandable adult pancreatic cells, CD9^+^, PROCR^+^ IPCs, that give rise to a subset of RGS16^+^ IPC clusters with the capacity to differentiate into glucose-responsive islet organoids *in vitro* and *in vivo*. Using this model, we further explored the mechanisms governing islet lineage differentiation by interrogating developmental transcriptional programs activated during IPC-to-organoid transition.

Differentiation of IPC clusters into insulin- and glucagon-producing islet organoids could be driven by ISX9, a small molecule previously shown to preserve beta-cell identity through chromatin modulation. ISX9-induced differentiation was dependent on calcineurin (CN) signaling and binding of NFATC2 to the RFX6 and NEUROD1 gene promoters. This promoter association was accompanied by CN-dependent recruitment of the histone acetyltransferase p300 and displacement of histone deacetylases HDAC1-3, suggesting that transcriptional activation is regulated, at least in part, by site-specific chromatin acetylation. Pretreatment with the HDAC inhibitor ITF2357 globally increased p300 enrichment on the RFX6 promoter and enhanced islet organoid differentiation.

Collectively, these data suggest that CN/NFATC2 contributes to selective epigenetic induction of the RFX6 gene to bypass the need for NGN3, a transcription factor essential during embryonic islet development (6, 7). In contrast, induction of the NGN3 promoter to enhance the potency of differentiation by HDAC inhibition occurred independently of CN activity or binding of NFATC2. These distinct mechanisms define critical signaling components required for inducing genes that initiate differentiation of IPC clusters into islet organoids.

Importantly, induction of the RFX6 and NEUROD1 genes permitted expression of genes in islet organoids required for both terminally differentiated beta cells and alpha cells. The resulting mature islet organoids expressed and released insulin and glucagon in a glucose-regulated manner. The islet organoid beta-like cells activated canonical insulin transcription factors, including PDX1, NEUROD1, and MAFA, and exhibited NFATC2 binding to the insulin promoter in response to glucose and GLP-1, recapitulating signaling pathways used by mature beta cells to maintain glucose responsiveness.

Limitations of our study include the exclusive use of male mice, which does not account for potential sex-specific effects in diabetes pathophysiology or treatment response. The frequency and anatomical distribution of IPCs in native human pancreatic tissue remain to be determined and warrant further investigation. In addition, the relatively small sample size limits statistical power and may constrain the generalizability of these findings.

*In vivo* studies were performed using expanded IPCs co-transplanted with marginal human islets to assess whether IPCs could enhance graft function under suboptimal conditions. Fully differentiated organoids were not transplanted in the present study due to current limitations in generating sufficient organoid mass to match standard human islet equivalents used in the transplantation model. The relatively modest magnitude of glycemic improvement likely reflects both limited cell dose and incomplete functional maturation at the time of transplantation. Future studies will focus on scaling IPC expansion and differentiation, as well as optimizing protocols to increase islet organoid graft mass and functional output prior to transplantation.

The anatomical and physiological basis of the transient residual glycemic effects observed following nephrectomy was not directly determined. Ongoing investigations are evaluating whether IPC migration contributes to restoration of pancreatic function; however, the observed effects may also reflect indirect or host-mediated cellular mechanisms rather than sustained extra-graft insulin production.

Alpha cells play an important role in counterregulatory responses to hypoglycemia and in paracrine modulation of β-cell function. Although definitive confirmation of fully mature α-cell identity was not established, ISX9-induced IPC-derived islet organoids demonstrated upregulation of ARX and PAX6, distinct regions of glucagon expression, and glucose-responsive glucagon secretion under low-glucose conditions. These findings support activation of α-like transcriptional and functional programs within differentiated clusters. However, the precise extent of α-cell maturation and spatial organization remains to be determined and may influence the overall functional integration of IPC-derived organoids.

Terminology describing three-dimensional endocrine aggregates varies across the literature and includes “pseudoislets”, “spheroids”, and “islet organoids”. The IPC-derived structures described here are self-organized, multi-lineage endocrine clusters that fall within the typical size range of isolated human islets (approximately 50–300 µm) and exhibit glucose-responsive hormone secretion. While complete recapitulation of native islet cytoarchitecture was not definitively demonstrated, these structures fulfill key functional and compositional criteria consistent with islet organoids. Further refinement of spatial organization and endocrine cell ratios may enhance architectural fidelity to native islets.

**Figure 8:**
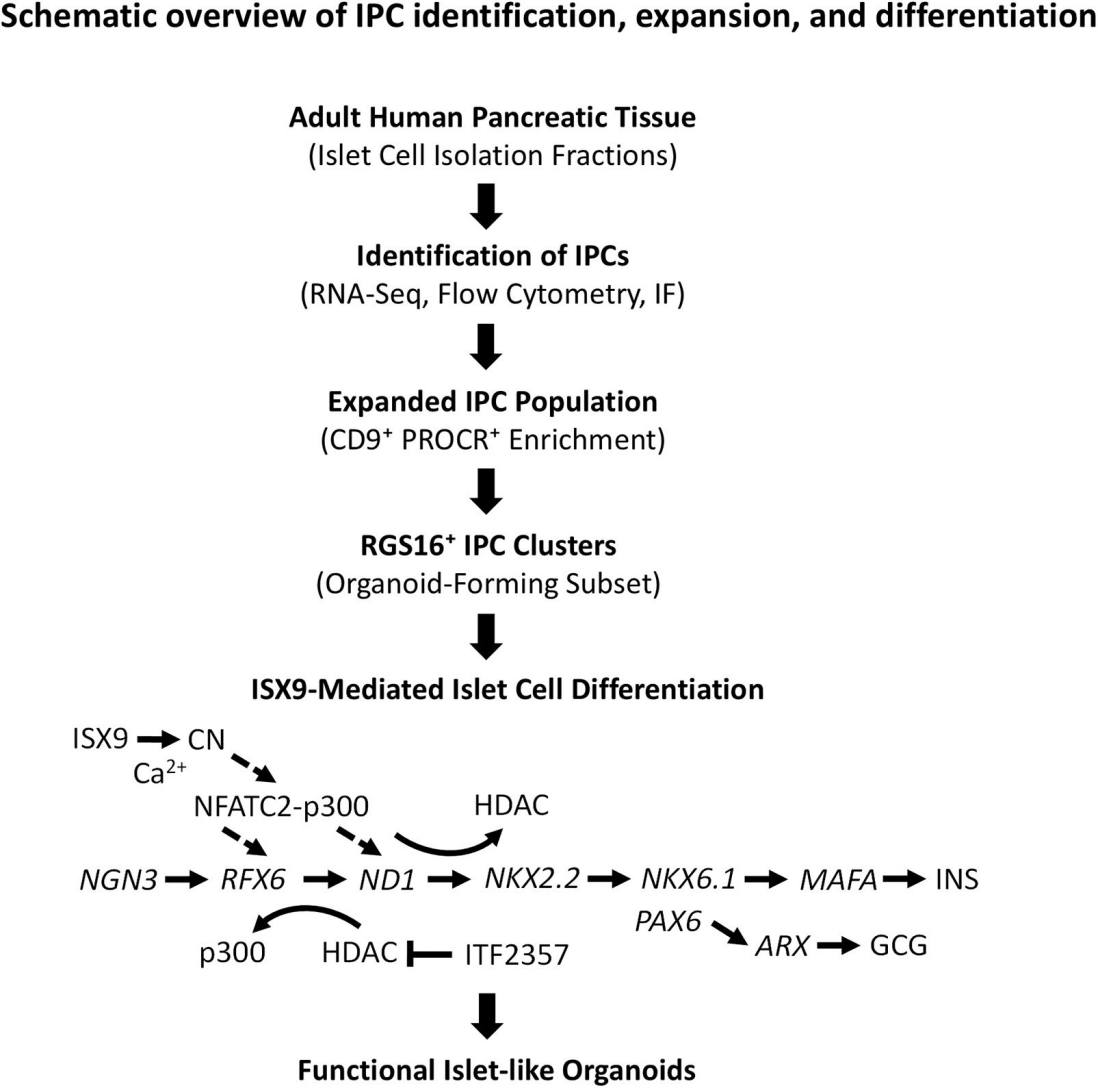
Schematic overview of IPC isolation, expansion, and differentiation. Adult human pancreatic tissue obtained from islet cell isolation fractions was expanded *in vitro* to generate a CD9^+^ PROCR^+^ IPC-enriched population. IPCs were identified using transcriptomic and phenotypic analyses (scRNA-seq, flow cytometry, and immunofluorescence). An RGS16^+^ organoid-forming subset was characterized within the expanded population. IPC clusters were subsequently subjected to ISX9-mediated differentiation. ISX9 treatment stimulated calcineurin (CN)/NFAT signaling, promoted NFATC2–p300 association and binding at RFX6 and NEUROD1 promoters, and induced downstream endocrine transcriptional programs (NGN3, RFX6, NEUROD1, NKX2.2, NKX6.1, MAFA), resulting in functional islet organoids.

Overall, these findings provide new insight to methodologies and mechanisms for expansion and differentiation of adult human IPCs into islet organoids (Figure 8). While recent advances have demonstrated the ability to derive beta cells from human pluripotent stem cells (1, 2, 93–95), no naturally existing adult islet progenitor cell has been definitively identified to selectively regenerate islet organoids in humans. We report that IPC clusters selectively express RGS16, an islet progenitor marker, and can be differentiated by ISX9 via CN/NFAT-mediated activation of *RFX6*. This process is enhanced by ITF2357, which facilitates chromatin remodeling by NFAT to induce activation of genes required for driving islet cell differentiation and maturation.

The identification of signaling pathways and transcriptional targets that govern adult IPC differentiation into functional islet organoids represents a critical advance toward regenerative medicine strategies for diabetes. These findings may enable the development of scalable, cell-based and pharmacologically directed therapies to restore islet endocrine function in individuals with pancreatic disease or diabetes.

## Data Availability

The datasets presented in this study can be found in online repositories. The names of the repository/repositories and accession number(s) can be found below: https://www.ncbi.nlm.nih.gov/, PRJNA1290648; SAMN49931282; SRS25765186.
