# Supplementary material for: Mechanisms inducing differentiation of adult islet progenitor-like cells into functional islet-like organoids
Source: Front Transplant. 2026 Feb 20;5:1740314. doi: 10.3389/frtra.2026.1740314 (PMC12963012; doi:10.3389/frtra.2026.1740314)
Supplement: Supplementary file 1 [file Datasheet1.pdf]

Suppl. Table 1

Human pancreas donor characteristics				
**Identifier/UNOS#**	Age	Sex	BMI	Ethnicity
**Donor_001**	65	Female	24.2	White
**Donor_002**	57	Female	23	White
**Donor_003**	45	Male	29.8	White
**Donor_004**	25	Female	35.5	Black
**Donor_005**	22	Male	33.2	Hispanic
**Donor_006**	29	Male	26.3	White
**Donor_007**	60	Male	26.7	Black
**Donor_008**	60	Male	28.7	Hispanic
**Donor_009**	46	Male	30.7	Hispanic

Suppl. Table 2

Markers used to identify cel types

Cell Type	Cell Markers
Acinar Cell	AMY2A, AMY2B, CPA1, PTF1A
Islet Cell	INS, GCG, IAPP, SST, PPY
Islet Progenitor Cell	PROCR, BMPR1A, RGS16, ISL1, NES
Immature Beta Cell	CD9, CD81, YAP1, MMP2, FOXO1, HK1
Beta Cell Disallowed	LDHA, SCL16A1, ACOT7, OAT, PDGFRA, CAT, IGFBP4, ZFP36L, ZYX, LMO4
Ductal Epithelial	KRT19, SOX9, CFTR, EPCAM, CDH1
MSC Positive	ENG (CD105), NT5E (CD73), THY1 (CD90), ITGB1 (CD29)
MSC Negative	PTPRC (CD45), CD34, CD14, ITGAM (CD11b), CD79A, CD19, HLA-DRA
Stellate Cell	ACTA2 (alpha-SMA), CYGB
Endothelial Cell	CDH5, PECAM1, VWF, CLDN5

Suppl. Table 3

Top cell type enrichment genes - PanglaoDB Augmented

Index	Name	P-value	Odds Ratio	Combined score
1	Pancreatic Progenitor Cells	0.00002316	11.70	124.85
2	Osteocytes	0.0001328	11.21	100.06
3	Kidney Progenitor Cells	0.0001837	10.42	89.64
4	Cardiac Stem And Precursor Cells	0.0002588	9.65	79.67
5	Adipocyte Progenitor Cells	0.0002807	9.47	77.44
6	Luteal Cells	0.0004124	8.68	67.61
7	Pulmonary Alveolar Type I Cells	0.0005111	8.26	62.60
8	Melanocytes	0.0005477	8.13	61.05
9	His Bundle Cells	0.001483	8.78	57.18
10	Embryonic Stem Cells	0.001913	6.07	38.01

Top cellular and tissue enrichment genes - CellMarker 2024

Index	Name	P-value	Odds Ratio	Combined score
1	Progenitor Cell Pancreas Human	0.0006796	67.67	493.57
2	Ductal Cell Pancreas Human	0.00006478	47.31	456.30
3	Plasma Cell Undefined Human	0.00009371	41.00	380.29
4	Germinal Center B Cell Undefined Human	0.001085	50.74	346.39
5	Marginal Zone B Cell Undefined Human	0.001085	50.74	346.39
6	Astrocyte Cortex Human	0.001581	40.59	261.80
7	Cholangiocyte Liver Human	0.001581	40.59	261.80
8	Neural Stem Cell Brain Human	0.0002274	29.28	245.59
9	Epithelial Cell Stomach Human	0.002166	33.82	207.50
10	Epithelial Cell Kidney Human	0.002491	31.22	187.17

Suppl. Table 4

A) Top upregulated signaling systems - GSEA Human Molecular Signatures Database (MSigDB)

Index	Name	P-value	Odds Ratio	Combined score
1	Epithelial Mesenchymal Transition	0.0005070	6.48	49.19
2	p53 Pathway	0.0005070	6.48	49.19
3	Pancreas Beta Cells	0.01706	10.67	43.42
4	IL-2/STAT5 Signaling	0.003253	5.35	30.62
5	TGF-beta Signaling	0.02993	7.79	27.33
6	Coagulation	0.03200	4.53	15.59

B) Top upregulated biological processes - Gene Ontology (GO) Molecular Function

Index	Name	P-value	Odds Ratio	Combined score
1	Aldehyde Dehydrogenase (NAD+) Activity (GO:0004029)	0.002166	33.82	207.50
2	BMP Receptor Activity (GO:0098821)	0.02475	50.24	185.84
3	Pyruvate Transmembrane Transporter Activity (GO:0050833)	0.02475	50.24	185.84
4	Pyruvate Transmembrane Transporter Activity (GO:0050833)	0.02475	50.24	185.84
5	Secondary Active Monocarboxylate Transmembrane Transporter Activity (GO:0015355)	0.02475	50.24	185.84
6	Sphingosine N-acyltransferase Activity (GO:0050291)	0.02963	40.19	141.43
7	G Protein-Coupled Acetylcholine Receptor Activity (GO:0016907)	0.02963	40.19	141.43
8	G Protein-Coupled Neurotransmitter Receptor Activity (GO:0099528)	0.02963	40.19	141.43
9	Carbohydrate:Proton Symporter Activity (GO:0005351)	0.02963	40.19	141.43
10	MHC Class II Protein Binding (GO:0042289)	0.02963	40.19	141.43

Suppl. Fig. 1.

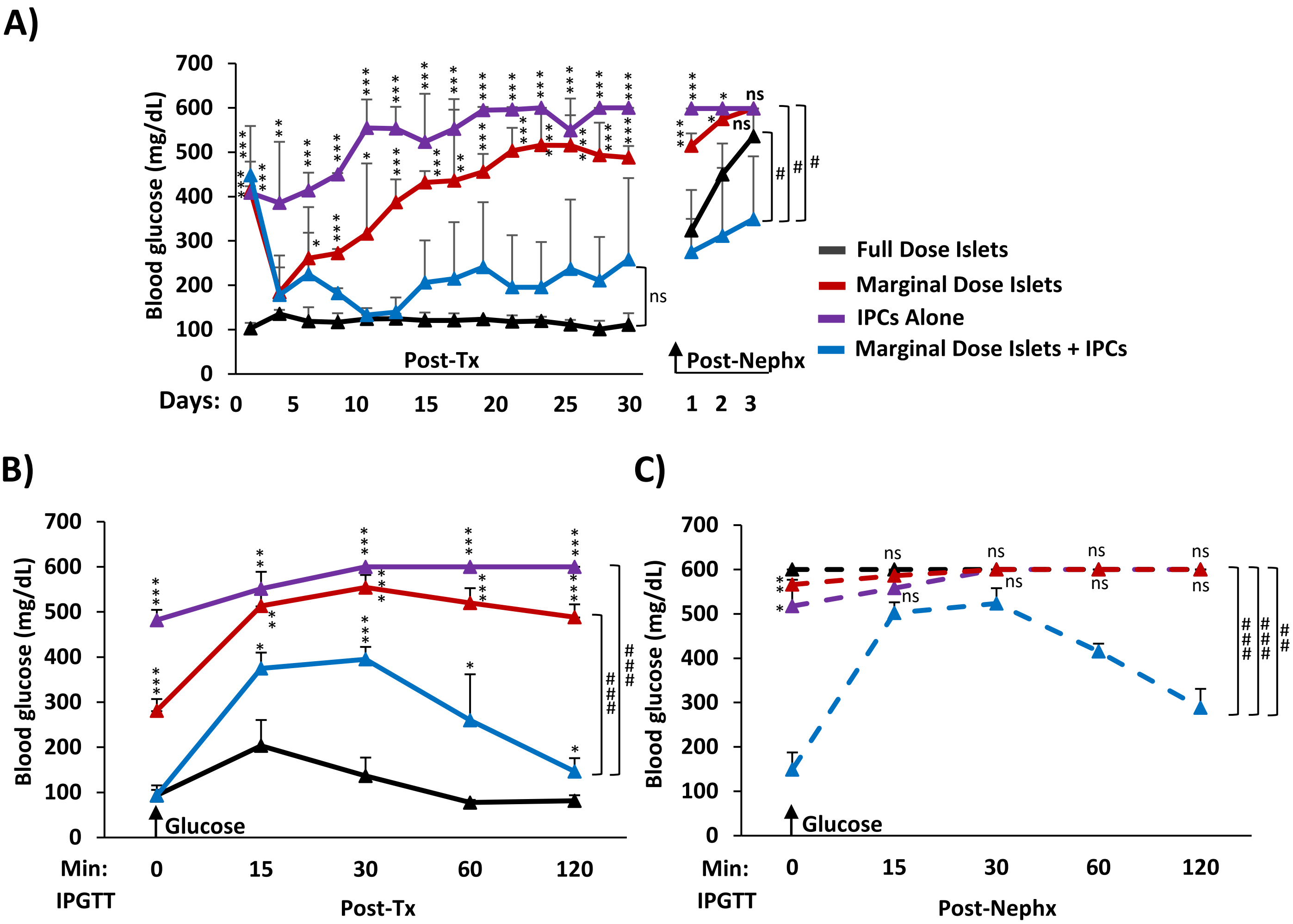

Suppl. Fig 1. Assessment of human IPC effects on islet cell function in an STZ-diabetic nude mouse bioassay. Longitudinal non-fasting blood glucose over 30 days post-transplantation and 3 days post-nephrectomy (A) and 1-hour intraperitoneal glucose tolerance testing (IPGTT) 30 days post-transplantation (B) and 3 days post-nephrectomy (C). Graphed values are expressed as mean ± SD. Asterisks above data points indicate statistically significant differences relative to marginal islet–only controls at the corresponding time point (ns, not significant;* p < 0.05, ** p < 0.01, *** p < 0.001). Hashtags adjacent to brackets indicate statistically significant differences between indicated treatment groups (# p< 0.05, ## p< 0.01, ### p < 0.001).

Suppl. Fig. 2.

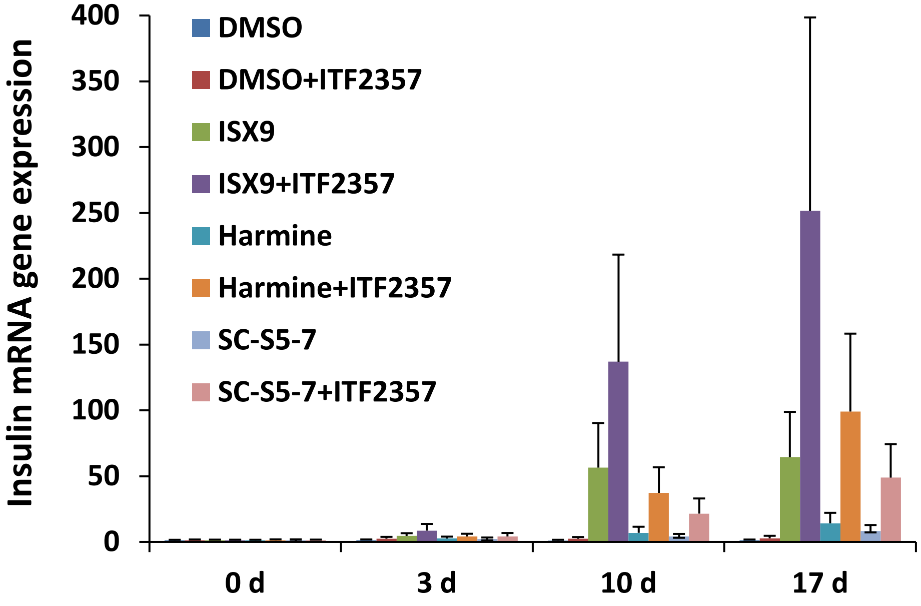

Suppl. Fig 2. Comparison of treatments to induce insulin gene expression in islet organoids. IPC clusters were treated with DMSO control, ISX9, Harmine, and differentiation medium SC-S5-7 alone and in combination with ITF2357 for 0 d, 3 d, 10 d, and 17 d.

Suppl. Fig. 3.

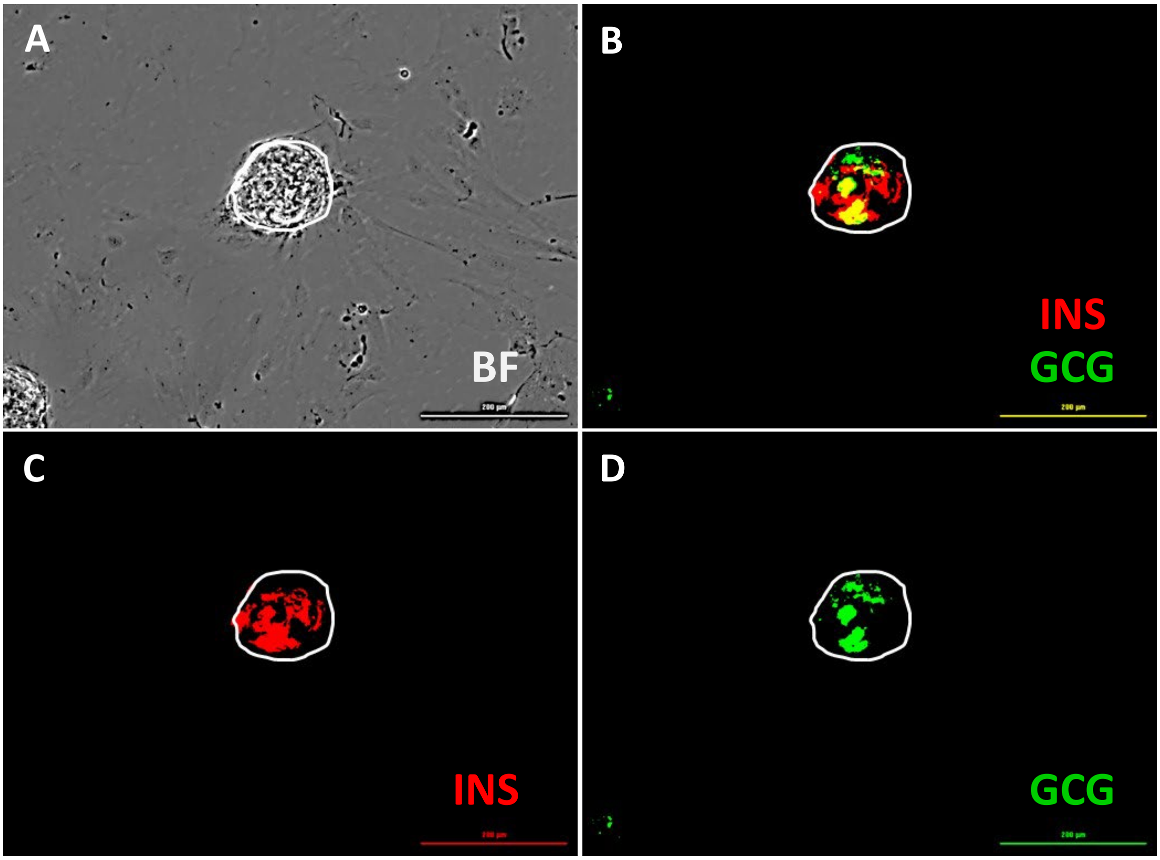

Suppl. Fig 3. Co-expression of insulin and glucagon in differentiated islet organoids. Representative full-field micrograph images of an islet organoid and IPC monolayer following 14-day ISX9-mediated differentiation including (A) brightfield image (BF); immunofluorescence images of (B) merged insulin (INS) (red) and glucagon (GCG) (green); (C) insulin alone; and (D) glucagon alone. Single-channel views are shown to demonstrate marker-specific signal distribution. Scale bars, 200 µm.
